# Digital depression screening in HIV primary care in South Africa: mood in retroviral + application monitoring [MIR + IAM]

**DOI:** 10.1017/gmh.2018.35

**Published:** 2019-01-28

**Authors:** R. V Passchier, S. E Owens, M. N Wickremsinhe, N. Bismilla, I. D Ebuenyi

**Affiliations:** 1 Department of Psychiatry and Mental Health, University of Cape Town, Cape Town, South Africa; 2 U.S. Department of Health and Human Services, Office of Minority Health, Washington, USA; 3 Nuffield Department of Population Health, University of Oxford, Ethox Centre, Oxford, UK; 4 Department of Anaesthesiology, University of Witwatersrand, Johannesburg, South Africa; 5 Athena Institute, Amsterdam Public Health Research Institute, Vrije Universiteit Amsterdam, Amsterdam, The Netherlands

**Keywords:** eMental Health, global mental health, HIV/AIDS, major depressive disorder, screening, primary care

## Abstract

**Background.:**

Integrating mental health care into HIV services is critical to addressing the high unmet treatment needs for people living with HIV and comorbid major depressive disorder. Introducing routine mental health screening at the primary health care level is a much needed diagonal approach to enhancing HIV care. In low-resource settings with a shortage of mental health care providers, eMental Health may provide a novel opportunity to attenuate this treatment gap and strengthen the health system.

**Objective.:**

To conduct formative health systems research on the implementation of routine depression screening using a digital tool – Mood in Retroviral Positive Individuals Application Monitoring (MIR  +  IAM) – in an HIV primary care setting in South Africa.

**Methods.:**

A Theory of Change (ToC) approach was utilised through individual and group session interviews to design an intervention that is embedded in the local context. Ten experts and local stakeholders were selected from the UK and South Africa. Data were analysed thematically using Atlas.ti to identify interventions, assumptions, barriers and facilitators of implementation.

**Findings.:**

The participants considered digital depression screening in HIV care services relevant for the improvement of mental health in this population. The six main themes identified from the ToC process were: (1) user experience including acceptability by patients, issues of patient privacy and digital literacy, and the need for a patient-centred tool; (2) benefits of the digital tool for data collection and health promotion; (3) availability of treatment after diagnosis; (4) human and physical resource capacity of primary health care; (5) training for lay health care workers; and (6) demonstration of the intervention's usefulness to generate interest from decision-makers.

**Conclusion.:**

Digital depression screening coupled with routine mental health data collection and analysis in HIV care is an applicable service that could improve the mental and physical health outcomes of this population. Careful consideration of the local health system capacity, including both workers and patients, is required. Future research to refine this intervention should focus on service users, government stakeholders and funders.

## Introduction

### HIV and depression in sub-Saharan Africa

People living with HIV (PLWHIV) experience mental disorders and psychological distress at a two- to three-fold increased rate compared with the general population (Olatunji *et al*. 2006; Chibanda *et al*. 2014). Of these, major depressive disorder (MDD) is the most common (Dubé *et al*. 2005), with a reported prevalence ranging from 11% to 38% in sub-Saharan Africa (SSA) (Gaynes *et al*. 2015). PLWHIV who are also living with comorbid MDD can experience impaired psychosocial functioning and quality of life (Preau *et al*. 2008; Carrico *et al*. 2011; Chen *et al*. 2013). Furthermore, comorbid MDD has been associated with reduced motivation to seek health care, impaired initiation and adherence to treatment, and increased mortality in PLWHIV (Safren *et al*. 2001; Cook *et al*. 2004; Pence, 2009; Mayston *et al*. 2012; Rane *et al*. 2018). Despite the abundance of evidence, MDD remains underdiagnosed and undertreated in PLWHIV (Freeman *et al*. 2005; Seedat *et al*. 2008; Kagee & Martin, 2010; Pence *et al*. 2012; Marinho *et al*. 2016; World Health Organisation, 2017). The expansion of HIV treatment initiatives into low- and middle-income countries (LMICs) of SSA, a region with an estimated 25.5 million people infected with HIV who represent 69% of global prevalence (UNAIDS, 2016), provides an opportunity to integrate mental health services into existing programmes (Freeman *et al*. 2005; Collins *et al*. 2006).

### Integration of care

Integrated care for coexisting illnesses, especially physical and mental disorders, has proven to be practical and more cost-effective than separate disease care (Katon *et al*. 2010; Jenkins *et al*. 2011). Leveraging already existing HIV treatment facilities and programmes to integrate mental health care would expand the resource base, awareness and management of mental disorders (Freeman *et al*. 2005). In fact, integrating mental health into HIV services was recommended by the very first global HIV treatment initiative, ‘Treat 3 Million by 2005’ (‘3 by 5’). The ‘3 by 5’ initiative acknowledged that a successful HIV intervention programme *must* include the assessment of mental disorders as part of the normative service (WHO, 2003; Freeman *et al*. 2005). Screening for comorbid MDD in routine care is essential to mental and physical health outcomes for PLWHIV. Treatment of MDD is one of the best interventions for reducing adherence attrition that often accompanies clinical depression in PLWHIV (Krumme *et al*. 2015). However, many HIV services have taken on a vertical nature, with clinics solely focused on HIV and separated from all other treatments (Mahomed *et al*. 2014). This has resulted in inefficiencies in the health care delivery system as well as a missed opportunity to strengthen not only HIV services but also to promote mental health in all primary care services (Freeman *et al*. 2005; Mahomed *et al*. 2014).

### eMental Health

The shortage of providers trained to deliver mental health screening and treatment in primary care, particularly in LMICs (Saxena *et al*. 2007; Naslund *et al*. 2017), calls for creative solutions to be implemented. Task shifting – training non-physician healthcare workers to perform tasks traditionally undertaken by physicians – when also accompanied by appropriate health system re-structuring, is a potentially effective and affordable strategy for improving access to health care (Hongoro & McPake, 2004; Callaghan *et al*. 2010; Joshi *et al*. 2014). However, in LMICs with substantial staff shortages and failing health systems (Callaghan *et al*. 2010), non-physician healthcare workers are overburdened and may not be able to take on more responsibility (Calpin-Davies & Akehurst, 1999; Ojikutu, 2007; Van Rensburg *et al*. 2008). Therefore, traditional task shifting alone will not solve human resources problems in HIV services (Callaghan *et al*. 2010). In areas with an absolute shortage of all levels of staff, shifting mental health screening to *technology*, assisted by lay health care workers (non-medical staff) (Naslund *et al*. 2017), may make screening and early detection of mental health conditions more feasible. The use of information technology to improve access to and the delivery of mental health care is known as eMental Health (Wise, 2017; Wozney *et al*. 2017).

Computerised neuropsychological assessment devices (CNAD) are being increasingly used for large-scale eMental Health screenings both in high- and low-income settings (Bauer *et al*. 2012; Tomita *et al*. 2016). A CNAD is an instrument that utilises a digital interface instead of a human examiner to administer, score or interpret tests of brain function or neurologic health and illness (Bauer *et al*. 2012). For example, a study conducted in South Africa used a text-messaging application to successfully screen for depression in refugees (Tomita *et al*. 2016). CNADs have several advantages over traditional screen practices, including increased portability, decreased administration time and minimised examiner influence on participant performance (Bauer *et al*. 2012). However, CNADs should be viewed as a ‘resource’ for health workers, rather than as a ‘substitute’ (Bauer *et al*. 2012) and should not undermine the need for human resource development in mental health.

To the authors’ knowledge, considerations around implementing a digital screening tool to effectively screen and reliably diagnose depression in PLWHIV in a LMIC context have not been examined. A Theory of Change (ToC) approach was utilised as an evidence-based tool to assess the viability and acceptability of integrating an eMental Health screening intervention into an HIV primary health care settings in South Africa.

## Method

### Study design

This formative study, using the ToC framework, was conducted through individual interviews and a group session. ToC is a qualitative method of research used to describe the causal pathways through which a programme is hypothesised to have an effect (Breuer *et al*. 2014). The workshop and interviews had three aims: (1) to conceptualise a contextually relevant and informed digital screening tool as an intervention; (2) to initiate buy-in from key stakeholders; and (3) to construct a ‘ToC map’ as a visual depiction of the pathways to change (Breuer *et al*. 2014). In this study, digital depression screening for HIV-positive patients was referred to as Mood in Retroviral Positive Individuals Application Monitoring (MIR  +  IAM).

Ethical approval was obtained from the Human Research & Ethics Committee of the University of Witwatersrand, South Africa (Ref: M16067). Written informed consent was obtained from all study participants. Experts for individual interviews were drawn from the London School of Hygiene & Tropical Medicine and the Institute of Psychiatry, Psychology and Neuroscience, King's College London. Ethical approval to conduct ToC interviews and collect professional opinions from academic staff was not required by their ethics committees. Permission was obtained through email contact with the head of department at the respective institutes.

### Study setting and population

A ToC is developed in consult with key stakeholders and experts in workshops or interviews (Mason & Barnes, 2007). Stakeholders are individuals who might be involved in the implementation of the intervention or who might be affected by the intervention (Breuer *et al*. 2014). The selection criteria for stakeholders included HIV health care service providers and working professionals or academic researchers in the fields of mental health, HIV, health systems or digital technology. The group ToC session was facilitated by RVP and NB in a staff room at the HIV clinic in Charlotte Maxeke Johannesburg Academic Hospital, South Africa. Permission was granted by the clinical head of department and all clinic staff were invited to attend the session. The individual interviews were conducted telephonically or face-to-face by RVP in a private meeting space. Individual participants were selected on the basis of global expertise in the respective fields of global mental health, health systems, digital technology and mental health in HIV.

### Data collection

The group ToC session was conducted for an hour and each individual interview was conducted for 30 min. Facilitators used the basic concept of a digital depression screening tool to initiate the ToC discussion. The discussion started with the presumed impact of the intervention and worked backwards to cover all the domains of the ToC including long- and short-term outcomes, treatment, identification, resources and capacity. As the discussion moved through the ToC pathway, participants were asked to identify rationales, assumptions, interventions and possible methods of evaluation (indicators) for MIR  +  IAM. The individual interviews addressed the same domains. However, because individual interviews were only conducted for 30 min, questions were directed towards the interviewees’ expertise. Online Supplementary Appendix 1 describes specific questions used during each interview. Data saturation was achieved when no new themes emerged from the individual or group interviews and ToC (Fusch & Ness, 2015). Data were collected through process documentation, including participant lists, minutes and physical notes used to plot participant ideas.

### Analysis

Field notes were transcribed, imported into Atlas.ti version 7.5.18 (Friese, 2013) and analysed using a thematic approach. A list of *a priori* codes, derived from the conceptual framework of the ToC, were used to initially categorise the data. Later, these codes were refined into sub-themes or expanded into larger themes (Gibbs, 2008). A chart was constructed with the final themes from the participant views. The ToC map was constructed using the chart to justify the pathway to effect. The initial coding was developed by IDE and RVP independently; differences were resolved through discussion and finalised by co-authors.

## Results

### Characteristics of study participants

Individual interviews were conducted with study participants in the UK from September to October 2016. These participants included one academic in the field of health systems, one mental health and HIV researcher, one psychiatrist working in the field of depression in LMICs with a focus on PLWHIV and, one expert in digital technology. The group ToC workshop took place in March 2017. Six clinic staff attended the session including two HIV care nurses, two infectious disease resident physicians and two infectious disease consultant physicians.

### ToC pathway to change: rationale, outcomes and impact

**Fig. 1.**
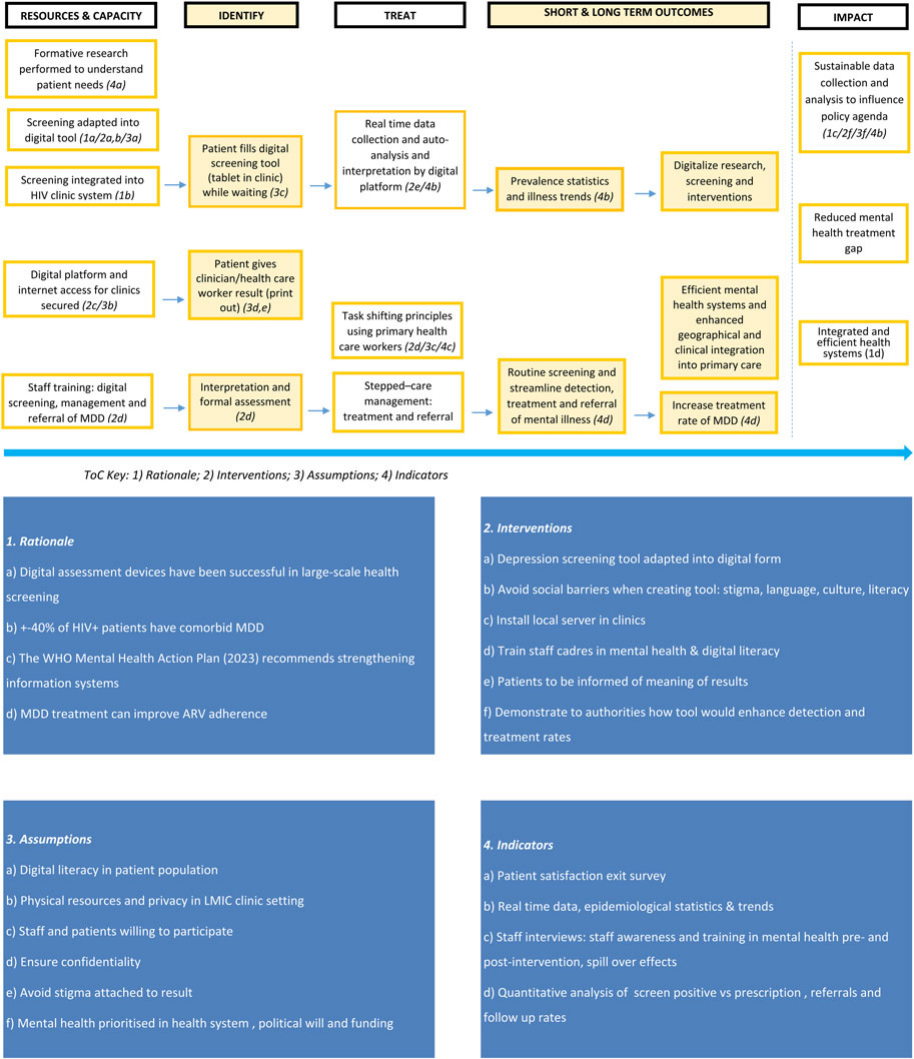
Theory of Change.

The ToC map summarises our findings and plots the pathway to change (Fig. 1). The study participants unanimously agreed that, if adopted and implemented to scale, MIR  +  IAM has the potential to improve the diagnosis and treatment of depression in PLWHIV in South Africa. The long-term outcome would integrate screening, diagnosis and treatment of depression into HIV clinics. Early detection of depression can have a significant impact on successfully treating depression, HIV and other chronic comorbidities by streamlining the process of entering mental health systems and enhancing geographical and clinical integration of treatment. The participants identified a strong rationale for the intervention because MIR  +  IAM targets a vulnerable population with a known elevated risk for and prevalence of depression. Health care practitioners in the group ToC acknowledged that medical practice should keep up with the digital trends of other professional fields. Furthermore, mobile and digital device use in Africa is high, indicating potential acceptance of a digital intervention in SSA. Little stigma exists within the HIV clinic between patients, thereby making the clinic a practical setting for a screening intervention. The six themes that emerged from the ToC framework are presented in Table 1 and discussed below.

**Table 1:** ToC Framework & Themes

ToC Framework	Main Theme	Sub Theme	Stakeholders and Expert Views
*Identify Population/ Assumptions/Indicators*	*User Experience*	*Target Population*	Adults and Adolescence
			Newly diagnosed patents may be high risk of MDD
			Consider excluding stage III patients due to associated cognitive decline
			Formative research: patient focus groups to discover what patients want to know at different stages of illness; what information do they most desire as a PLWHIV
		*Privacy & Data Safety*	Unidentifiable data: No name or patient number to be entered into the program
			Screening to be explicit in its purpose and results vs Screening to use symbols and colours for interpretation by staff
			The fear of personal data on the internet
			Privacy compromised e.g. moving queues, few private rooms available
			Safe website and measures to demonstrate data safety and confidentiality
			Privacy settings can be managed centrally
			Willingness of patients to take survey and submit information digitally
		*Acceptability*	Depressed patients may be less likely to participate due to higher stressors or decreased motivation)
			Avoid stigma- no distinguishing factors between mental health referrals and other patients
			Digital literacy and language spoken by patients must be considered and integrated into the tool
			Qualitative indicators: Patient interviews- satisfaction and perceptions of intervention fitted with patient expectations, priorities and needs
			Extend clinical interaction beyond the consultation
			Demonstrate that digital screening tool would enhance care to achieve better patient buy in
			Use an MDD screening tool validated in the culture and language of the region
			When designing the digital tool, the starting point is the patients need- adapt the technology to revolve around patients need
			Tool could be patient specific to allow for continuation of care and follow up
			Result of screening should be physically recorded (eg. Printed slip or written by nurse in file)
			Do not rely on patients to remember their score result
			Sensitivity to the reactions of patients to screening questions
*Resources/ /Outcomes/ Indicators*	*Optimizing the Digital Tool*	*Data Collection*	Real time mental health population data
			Follow up and monitoring system for patients who begin MDD treatment e.g. retention, improvement in physical and mental outcomes
			Generate better understanding of how to communicate mental health information and provide effective treatment to people with chronic disease in LMIC
			Understand what information is required by patients and how it can be provided
			Group data aggregated to create statistical picture of MDD in HIV primary care
			Tool could amalgamate individual patient data
			Same digital system could be introduced throughout stepped care allowing follow up
		*Health Promotion*	Expand beyond screening tool: provide education, access to electronic health records
			Mental health education to be incorporated into daily clinic health talk
			Mental health education to be incorporated into screening tool
			Information campaigns to promote intervention
			Social support made available for social issues that arise
			Broader mental health screening e.g. PTSD, substance abuse, anxiety etc.
			Expand program to include screening for lifestyle diseases e.g. hypertension and diabetes
			Key target is screening: Rate of detection of MDD should increase vastly since no previous method was in place
			Screening results vs recorded cases in clinic notes vs prescription vs dispensing of medication
			Requires at least six months to generate useful outcomes
*Treatment / Impact and Outcome*	*Access to Treatment*	*Principles of Justice*	Must provide treatment and a referral pathway after screening
			Patient centred treatment – requires formative research with service users
			Medication should be available at clinic
			Patients to have psychiatric/psychology follow up
			Doctors or nurses should diagnose and immediately initiate treatment for MDD at the HIV clinic
			Stepped care: Patents treated according to level of care required e.g. Referral to specialist for emergencies (eg. suicide ideation)
			Management protocol available
*Resources / Assumptions*	*Capacity*	*Physical Resources: Digital Tool*	Digital tool could be provided by the clinic e.g. tablet fixed to a stand
			Patients could access the tool using their mobile device
			Private area provided to complete screening tool
			Printing device to print hard copy of results
			Also consider paper based screening
			Web-based platform: ‘responsive’ platform which allows a browser in any device to access the screening tool
			Central control to manage and update the program at any time
			Real time feedback from patient screening results and clinic mental health data analysed from the ‘backend’ of the program
			Data collected on the device can be uploaded manually onto the centralised system at a different location where internet is available
			Cloud System: electronic data collection for epidemiological key indicators e.g. vulnerable groups, surveillance, data analysis and follow up (process indicators) using an automated system
			Installation of local server for remote clinics
			Storage for offline devices: size can vary depending on the scale of the data
			Recording screening results in the clinic is similar to already existing standard practice of recording and reporting viral load and CD4 counts of patients which is routinely used for research and statistical purposes
			Ensure psychotropic medication available at HIV primary care level
		*Technical Challenge*	The application can continue to be used and data internally stored when no internet connection is available
			Internet availability (no wifi at local clinics) /irregularity may obstruct its use
			Poor facilities for screening
			Safety of equipment in clinic
			Different clinics have varied levels of resources and capacity for implementation
			Device may be stolen from clinic
			Patients may not have mobile phones and data/wifi connection to access screening
			Data anonymized
			Digital tool must add value beyond a paper based screening tool
		*Human Resources: Health Care Workers and Technical Experts*	High staff work load
			Intervention requires human resources (nurses to help patents use tool)
			Who will deliver training to staff
			Hesitation to initiate MDD treatment due to fear of interactions with ART
			Digital tool can be developed in a relatively short space of time (+- 5 days for the client facing program and +- 10 days for the backend of the program
			Physical illness takes priority
			Stigmatization can result in poor treatment within services
			Insensitivity to other patients if workers spend more time with patients who screen positive
		*Social Resources: Organizational Culture*	Mental health is not currently on the health systems agenda
			Cultural differences between sectors e.g. clinic vs mental health professionals vs technical engineers- difference in schedules, resistance to change, personal objectives
			Local perceptions of MDD and digital screening must be considered
*Intervention/ Assumptions/ Indicators*	*Training*	*Task Shifting*	Use task shifting principles
			Staff member assist patient in completing the digital screening tool
			Morning health talk to incorporate explanation on the screening tool and health education on MDD
			The intervention should fit the needs of the service providers (not only service users)
			Staff trained to help patients use digital screening tool
		*Skills*	Staff trained to diagnose and treat MDD
			Digital literacy of staff
			Different level of staff training
			Multiple staff cadres need to be trained e.g. doctors, nurses, counsellors
			Ongoing supervision required after training and task shifting staff
			Quality of screening needs to be maintained
			Staff interviews- gather opinions, suggestions, identify resistance and resolution to problems, spill-over effects
			Quantitative Indicators: Symptom relief, psychiatric admissions, ART adherence, follow up rates
*Indicators*	*Generating Interest*	*Demonstrating Usefulness*	Generate a conversation, create awareness and ‘plant the seed of interest’
			Trust building: create a space for open communication and capacity to collaborate
			Engage community, family support and NGO's to link up
			Generate interest from the ‘top’
			A senior leader needed to ’champion’ the cause and have regular contact with the intervention and accountability
			Communication between sectors both formal and informal e.g. weekly reviews of the intervention process with clinic staff
			Commitment from intermediate level managers
			Recognise who holds the power to translate and activate intervention on a regional level
			Demonstrate the usefulness of the concept to patients and stakeholders
			Use local evidence to demonstrate possible benefits
			Quantitative: Symptom relief, psychiatric admissions, ART adherence, follow up rates
*Outcomes/ Impact*	*Short Term Outcomes*		Screening and treatment of MDD
			Integration of treatment (HIV and psychiatric care)
			Pilot testing
			Political and funding buy-in
			Streamline process (referral) to enter mental health system
	*Long Term Outcomes*		Increased number of trained staff to deliver mental health care
			Increased acceptability of MDD treatment to staff and patients
			Scale up of intervention
			Improved metal and physical health in PLWHIV
	*Impact*		Improving mental health care in LMIC
			Use existing pathways of care which have established regular contact with patients, to implement mental health provision
			Improve implementation and intervention research in the field of HIV and MDD
*Rationale*		*Setting and population*	Medical practice should keep up with the digital trend of other fields
			Patients talk openly within the queue- there is little stigma within the clinic
			Africa has shown to have a large digital usage
			Target vulnerable population with known high risk and prevalence for depression

#### (1) Centring user experience: target population, acceptability and privacy

The participants in the group ToC suggested that the intervention could be targeted at both adults and adolescents, and that the intervention should consider high-risk groups such as newly diagnosed patients. Extensive formative research with service users is required to inform the intervention. This research could be conducted through patient focus groups to discuss user expectations of the intervention, preferred information delivered by the intervention, and user perspectives on entering mental health data into a digital tool.

The introduction of digital depression screening to the patients and their acceptance of the intervention is a rate-limiting step to this intervention. Firstly, it is essential that HIV patients with comorbid depression accept their HIV status and demonstrate willingness to access care. Secondly, according to the health system expert, patients would require basic digital literacy skills and must be willing to input their data into a digital survey. The interviews surfaced the assumption that, in order for MIR  +  IAM to have uptake, the tool is confidential and data-secure. This assumption rests heavily on the health care workers and the security protocols of the digital tool; this assumption could limit patients’ acceptance of intervention use. The digital technology expert recommended that data should be unidentifiable, requiring no name or patient number to be entered by the service user into the screening device. Lastly, family or caregiver support was recognised as pivotal to patients’ acceptance of the intervention. Thus, the starting point of the intervention should centre the patients’ needs, and technology ought to be adapted to fit those needs.

The screening tool should have the capacity to screen a patient successfully based on self-imputed data. Therefore, the MDD screening tool used must be validated in the culture and language of the region, and the technology built should be informed by service users. In regard to the presentation of a patient's results, two schools of thought arose. Firstly, some experts agreed that it might be beneficial to give results as a symbol (e.g. red fruit to symbolise high risk) or number, to avoid stigmatisation. Secondly, other experts acknowledged that often patients talk openly among themselves at the clinic, and deserved to receive immediate and explicit information about their screening result. Ultimately, rather than relying on patients to memorise their result, the participants in the group ToC agreed that results from the screening need to be physically recorded and added to patient's health records.

Lastly, adverse local cultural perceptions of MDD and digital screening (i.e. stigma) could be another barrier to implementation. The cultural and social differences between the patients and the other actors (e.g. nurses, clinicians, technical engineers) may impede acceptance of MIR  +  IAM. This includes social barriers such as poverty, language and digital literacy. In addition to the human and cultural barriers, barriers emanating from the illness experience (depression) might predispose the patients to higher levels of stress that may dampen motivation to accept the intervention.

#### (2) Optimizing the digital tool: data collection and health promotion

The digital technology expert suggested that the ‘backend’ of the tool [the technology that powers the components which enable the user-facing side of the website to exist (Wales, 2014)] should provide real-time data collection, auto-analysis and interpretation by a digital platform. This would provide current statistics to be used as key epidemiological indicators. Again, different views were expressed regarding patient data collection. Some participants thought the data should be anonymised and used as epidemiological statistics (e.g. prevalence trends), while others suggested that data on each patient be specific and followed up. Linking the digital tool with patient electronic health records would create a monitoring system for patient retention, treatment and outcomes, thus expanding the intervention beyond screening which could lead to improved continuity of care. Furthermore, participants described additional screening mechanisms for the digital tool, such as identifying those at risk for hypertension and diabetes.

Participants in the group ToC suggested that the incorporation of MDD screening into the HIV clinic could enhance health promotion, e.g. by introducing routine educational talks on MDD while patients wait (often for long periods) to be seen by a physician in the clinic. Furthermore, the digital platform opens an opportunity to provide primary illness prevention via virtual education incorporated into the screening tool. Creating an innovative digital tool that is patient-centred ultimately increases opportunities for health and mental health promotion among patients.

The health system expert recommended that the success of the screening tool can be assessed by quantitative analysis of the number of screen-positive results *v.* the number of prescriptions written and treatment dispensed. Additional indicators of successful implementation include symptom relief, admissions, treatment adherence, follow-up rates, satisfaction and patient perspectives of screening evaluated through patient exit surveys. Intermediate indicators may take up to 6 months to generate. Overall, an increase in detection of depression would be the most pivotal indicator of success as no previous method for detection was in place.

#### (3) Promoting access to treatment: principles of justice

The assumption that health care workers would be willing to educate, treat or refer a patient who screens positive for depression is integral to the uptake and success of the intervention. After screening positive, patients must have access to immediate MDD formal assessment and treatment by a trained member of staff (of any cadre). The provision of formalised detection and care of MDD is critical to the ethical principle of justice, where a screening tool should not be provided without adequate treatment available. The mental health and HIV researcher, as well as participants of the group ToC, recommended a stepped care method of treatment, with patients receiving a level of care matched to their individual needs (e.g. counselling, psychology, medication and/or referral). To facilitate this, a protocol with the correct pathways to treatment needs to be provided.

#### (4) Building capacity: physical, human and social resources

The need for physical resources surfaced through the interviews, including the digital tool itself, a method of recording results of the screening and space to conduct the screening. A private area, e.g. in the room where each patient has their vital signs recorded, will be required to conduct screening. The digital tool could be provided by the clinic, or patients could access the screening programme using their personal mobile devices. The psychiatrist suggested that a paper-based screening tool should also be considered as an option. The digital technology expert considered a digital tool which used a web-based platform to be the most practical resource because of its ‘responsiveness’, allowing users to access the screening tool with any device that has Internet access and for technicians to update the screening tool remotely. However, within the clinic, the digital tool should function regardless of Internet connection, and be able to store data until it can be uploaded onto a data-secure central server or ‘cloud’ system. Installation of the local server and storage capacity for the offline devices should be considered in remote areas. However, the digital technology expert explained that the amount of physical resources required would vary depending on the scale of the programme. A printing machine would be required to print results. This takes the burden of remembering their result off the patient and creates a hard copy for medical practitioners to keep a record of the patient's depression screening results. Practical barriers to consider in the context of a LMIC clinic include poor Internet availability (i.e. lack of access to personal data or Wifi) and storage capacity at local facilities. Furthermore, the lack of data safety may be a barrier since privacy within a crowded clinic can be compromised and digital tools may be liable to theft.

Another fundamental barrier highlighted was the heavy workload for staff in primary care. MIR  +  IAM is dependent on the assumption that primary health workers are capable of screening for depression and administering medications. Therefore, human resources would be needed for training and supervising lay health care workers to allow for successful task shifting. Additionally, the creation of a digital tool or ‘application’ requires expert skill and human resources. However, according to the digital technology expert, with a team of developers, the basic client face of the programme could be created in a very short span of time (about 5 days). The basic backend of the programme which manages security settings, credentials, user information, etc., could also be designed in a relatively short time (about 10 days).

Organisational culture was considered to be a possible barrier to implementation for multiple reasons. Critically, mental health is not currently on the health systems agenda, and a patient's physical illness often takes priority in time and resource restricted settings. An additional concern mentioned by the health systems expert was the possible tension created by cultural differences among various sectors involved with the digital screening tool. Programmers, medical staff and policymakers all have different agendas, schedules and methods of working on the tool. However, trust-building and the intersectoral collaboration is achievable if space to promote open communication, both informal and formal, is created between sectors (e.g. weekly reviews of the intervention process with clinic staff). Furthermore, community engagement, family support and local non-governmental organisations represent important social resources.

#### (5) Training: task shifting

Because MIR  +  IAM would be integrated within an existing clinic, its success would depend on health care workers as the primary human resource. One of the main barriers highlighted was the heavy staff workload in primary care. Principles of task shifting could be used to allow multiple cadres of health workers from the clinic to serve as human resources for the implementation of the intervention. Nurses were identified as the most feasible actors to implement the intervention. Multiple cadres of staff could be trained to use the digital screening tool, in order to better diagnose and treat MDD, followed by ongoing clinical supervision. The psychiatrist noted that this would require task shifting principles, again informed by formative research, to ensure the intervention fits the needs of the service providers. Staff interviews would also generate useful indicators of staff interest, workload perceptions and attitude about work. The willingness of the health care workers serves as a key indicator of success. This would be demonstrated in the agreement between screening results and trends in depression treatment of identified patents. According to the psychiatrist, the tool could be used to generate statistics on staff performance by comparing screening, referral and treatment records. The impact of such analyses may improve implementation and intervention research in the field of HIV and MDD.

#### (6) Generating interest: demonstrating usefulness

Both the mental health and HIV researcher as well as the health systems expert noted the importance of generating conversation, creating awareness and planting the seed of interest in the health workers through appropriate communication channels. Securing interest from the ‘top’, i.e. government health agencies, would be just as vital as gaining commitment from intermediate-level managers. Ideally, a senior leader would ‘champion’ the cause, take accountability and regularly inquire about the implementation of the intervention. Practical outcomes surfaced in the interviews included political and funder buy-in for the initiation of a pilot study and future scale up.

Outcomes of the intervention – measurable by data generated on clinical improvement and prevalence statistics – could demonstrate usefulness and encourage interest in the intervention. Such data may include short-term outcomes, e.g. symptom relief, admission, treatment adherence and improved care/satisfaction, or long-term outcomes, e.g. suicide prevention and adherence HIV treatment. Data collected by MIR  +  IAM could lead to overall improvement on health indices as a result of information generated by the screening tool. The mental health and HIV academic expert suggested that the data gathered could be used in the future planning of health care for patients with both depression and HIV. These data could also provide insight on the patients’ perception of services and care for further formative research.

## Discussion

This study aimed to conduct formative mental health systems research on the implementation of routine depression screening, using a digital platform, in HIV primary care in South Africa. The research covered expectations of impact, outcomes, interventions, resources, assumptions, indicators and rationale through a ToC framework. The research participants determined that using a digital screening tool to identify patients with MDD in HIV care services is relevant for the improvement of mental health in this population. The themes highlighted through the ToC process focused on several key areas: (1) user experience with the digital tool, MIR  +  IAM, including acceptability by patients; (2) utilisation of the tool for data and educational purposes while addressing the barriers of confidentially and privacy; (3) access to treatment; (4) resource capacity; (5) training through task shifting; and (6) generation of interest in the intervention.

User experience is fundamental to the success of the digital screening tool. Creating a well-designed, culturally and linguistically appropriate tool will help to ensure the engagement of patients who are inputting data on their own. Making the tool user-friendly for a variety of ages, languages and cultural perspectives may address the issue of acceptability across a diverse patient population. Expert participants emphasised the importance of patients receiving their results instantaneously and keeping the information in patients’ electronic records for future clinical reference. Storing patient data electronically can streamline information for more efficient coordination of care and treatment (Bates *et al*. 2014). Furthermore, providing immediate results to patients will promote autonomy to make decisions around their diagnosis. When providing results to patients, MIR  +  IAM can be used as a digital platform for educational purposes to inform service users about clinical depression and its impact on functionality. The digital screening tool could achieve not only point-of-care monitoring, but also could incorporate health promotion. Increasing mental health literacy in PLWHIV may reduce stigma around mental illness in this population and promote early detection of MDD symptoms in other patients. Combining screening and health promotion would improve operational efficacy of primary health systems and address issues such as patient flow. However, these findings need to be confirmed in larger samples or future systematic reviews. Adopting a holistic approach to prevention, detection and treatment would ensure that patients are treated as individuals and not as disease entities (Mahomed *et al*. 2014).

An additional use of the digital tool described during the study is data collection for several uses, including epidemiological research, records of mental health symptoms, and monitoring of treatment processes and outcomes for patients’ electronic records. The vertical nature of HIV services has resulted in a strong emphasis on the collection of data for HIV outcomes (Mahomed *et al*. 2014). Integration of mental health into the existing data culture and structure presents an enormous opportunity to ensure information for service improvement. Epidemiological research is particularly valuable in primary care because of the lack of policy-relevant information in LMICs (Tomlinson *et al*. 2009). This information could thus be used to generate interest from policymakers and funders and to advocate for mental health provisions. The use of the digital tool to gather and analyse such data aligns with the World Health Organisation's goals to strengthen information systems, evidence and research for mental health (World Health Organisation, 2013). Furthermore, streamline translation into policy, programmes and practice should be prioritised to realise change (Passchier, 2017). However, when collecting sensitive information from patients, protecting private and confidential information is critical to building and maintaining trust. The use of mobile or digital tools in health and mental health requires additional measures to secure sensitive information.

Training lay health workers to use eMental Health may reduce the mental health screening burden on health providers and make diagnosis and treatment more feasible in low-resource settings (Mendenhall *et al*. 2014; Naslund *et al*. 2017). As South Africa moves towards a universal health care system, technological tools will help to create a public health care infrastructure that is efficient, equitable and able to accommodate the needs of health care users (Fusheini & Eyles, 2016). There is a growing global consensus that health systems should evolve to harness the potential of technology (Wozney *et al*. 2017). However, leveraging digital tools and task shifting to readily screen and diagnose depression in HIV patients may dramatically increase the number of patients that will require mental health care. Therefore, screening should be recognised as only the first step, and access to treatment for those identified with MDD is essential (Krumme *et al*. 2015). We note the critical importance of aligning the implementation of MIR  +  IAM with health care administrators and government leadership to ensure that there are adequate mental health services available to address the clinical needs of patients (Padmanathan & De Silva, 2013). Therefore, social and physical resources are crucial considerations when assessing the feasibility of implementing digital tools in primary care settings. To successfully realise implementation, buy-in from mental health service providers, health care leadership and policymakers will be required. This buy-in could be won by generating interest through demonstration of the intervention's utility through pilot studies, data collection and evaluation.

Integration of mental health into primary health care services was outlined by the WHO Mental Health Action plan 2013–2020 (World Health Organisation, 2013; Marais & Petersen, 2015). The South African National Mental Health Policy Framework and Strategic Plan 2013–2020 (Department of Health Republic of South Africa, 2013) followed with a call for Integrated Chronic Disease Management (Asmal & Mahomed, 2014) and an emphasis on vulnerable populations, such as PLWHIV (Stein, 2014). There is a need to develop models for the delivery of mental health care based on the principles of affordability, acceptability and availability, as detailed by the ‘3 in 5’ Mental Health task team (WHO, 2003; Freeman *et al*. 2005; Mahomed *et al*. 2014). The use of technology in primary care settings is an opportunity to address the mental health gap in HIV primary care services. Using eMental Health may alleviate the burden of detecting and diagnosing depression in low-resource care settings that lack mental health practitioners. However, understanding and being responsive to the complexity of local realities, interests and contexts is essential for implementing a new strategy (Lehmann & Gilson, 2012).

### Limitations

Because ethical clearance for patient participation was not obtained, we were unable to include service users in the ToC. The lack of service user involvement is the most significant limitation of this study. Health system research should ensure that the policies and programmes that are put in place are responsive to the needs of health care users (Marais & Petersen, 2015). This key consideration was reiterated by the participants who called for further insight from service users. As Bishai *et al.* acknowledge, ‘people who own the problem can anticipate the most likely social obstacles to its resolution, and their participation is essential,’ (Bishai *et al*. 2015). A second group of key stakeholders that could have been included at this early stage was government officials from the Department of Health. These actors can exert valuable power over implementation of health policy, ranging from authoritative power – derived from hierarchy and budget control – to mid-level decisions to grant labour and training resources (Lehmann & Gilson, 2012). The value of engaging government stakeholders was noted as a key lesson in the PRIME ToC workshops, where the support of policymakers was necessary to add legitimacy to the workshops and increase the likelihood of implementation of the intervention (Breuer *et al*. 2014). In South Africa, where mental illness is a low priority and managerial and planning capacity to develop and implement mental health care plans is weak (Marais & Petersen, 2015), involvement of government stakeholders might increase the likelihood of intervention uptake in primary care services.

Another limitation is the limited participation by lower level staff in the group ToC workshop, potentially due to the hierarchical nature of service providers in the local health system (Breuer *et al*. 2014). Composition of workshops should strike a balance with staff members of different levels to promote open engagement among varied participants (Breuer *et al*. 2014). Seeking participation from high-level professionals through focused interviews allowed us to stratify participants and gain international opinion. However, because the interviews and the workshop were conducted separately, the development of the ToC map was fragmented. The final ToC was culminated at the discretion of the same researchers who ran the workshops. This may have resulted in biased view of the ToC with an overestimation of the viability and usefulness of the intervention. The final ToC map is therefore not entirely ‘owned’ by all the participants, as recommended by the Aspen Institute (Roundtable, 1997; Breuer *et al*. 2014).

Furthermore, this study is limited by the sample size of 10 participants. Input from a larger group of participants would have possibly elicited greater insights, and increased participation should be encouraged in future studies through vigorous reminders to staff. However, while the study's sample size was low, the quality and power of the information proved by the participants was high, as all participants were directly involved in fields relevant to the study's narrow aim of conducting formative research through a ToC approach (Malterud *et al*. 2016). Lastly, the interviews and group ToC were not audio recorded; therefore, the use of quotations from participants was not included in the results. Though the researchers took careful written notes, actual quotes would have increased the data quality of this study.

## Conclusion

Comorbid depression for PLWHIV has been the subject of extensive research; however, little action has been taken to address this unmet health care need. In SSA, where the burden of HIV and its associated morbidity is dangerously high, integrating mental health into HIV primary care services is, undoubtedly, a critical component of treatment for sustainable improved HIV outcomes. Formative research with key stakeholders and experts suggests that routine digital depression screening and mental health data collection through automated analysis is both a desirable intervention and a practical service that could improve the mental and physical health of PLWHIV. Careful consideration of the local health system's capacity, including facilities, health care workers and patients, is required. This evidence, though promising, is preliminary. Future research to refine the intervention should be informed by service users and should ensure buy-in from key decision-makers.

## Supplementary material

For supplementary material accompanying this paper visit http://dx.doi.org/10.1017/gmh.2018.35. click here to view supplementary material
