# Supplementary material for: Digital depression screening in HIV primary care in South Africa: mood in retroviral + application monitoring [MIR + IAM]
Source: Glob Ment Health (Camb). 2019 Jan 28;6:e2. doi: 10.1017/gmh.2018.35 (PMC6401371; doi:10.1017/gmh.2018.35)
Supplement: Supplementary file 1 [file S2054425118000353sup001.docx]

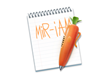
**APPENDIX 1**

Digital Depression Screening in HIV Primary Care in South Africa: Mood in Retroviral + Application Monitoring (MIR+AM)

**Question Sheet for Theory of Change Workshop/Focus Group Discussions/Interviews:**

Theory of Change (ToC) offers an approach to better understanding how, why and to what extent change happens as a result of the implementation of complex interventions and development programs. It can provide a rich process and impact framework to guide implementation and evaluation addressing barriers to implementation, and incorporating the rationale behind approaches taken and contextual influences (Mental Health Innovation Network, 2014). Questions adapted from Theory of Change Guidelines (De Silva et al., May 2015)

1. IMPACT

What is the impact or change in the real world that we want to achieve? -How will communities benefit?

1. OUTCOMES

-What long term, intermediate, and early outcomes are necessary to produce this impact?

1. INTERVENTIONS

-What interventions should be initiated to achieve intermediate and long term outcomes?

1. RESOURCES

-What resources are required to implement the intervention?

- What resources are required to maintain the contextual supports necessary for the intervention to be successful?

-How does the programme gain the commitment of those resources?

1. ASSUMPTIONS: BARRIERS & MEDIATORS

-What contextual conditions are necessary to achieve the intermediate outcomes?

-Describe the barriers to achieving intermediate outcomes

-Describe the facilitators to achieving intermediate outcomes

1. INDICATORS

-For each intermediate outcome, what indicator can be used to measure whether that outcome has been achieved. I.e. standard outcome measures of effectiveness. E.g. Change in PHQ-9 scores

-At what levels will indicators be measured? E.g. patient, primary health care, community

-How will each indicator be measured? E.g. Data collected electronically

-How much does the indicator have to change in order for us to claim that we have reached our intermediate outcome? E.g. 20% increase in diagnosis of depression in HIV clinic

-How long will it take to bring about necessary change in the target population? E.g. 3 months -When will each intermediate outcome be measured? I.e. Points of measurement. E.g. monthly measurements

Participants are invited to ask questions and to freely add any further points deemed relevant.


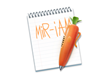
Digital Depression Screening in HIV Primary Care in South Africa: Mood in Retroviral + Application Monitoring (MIR+AM)

**The following questions were added at the request of University of Witwatersrand Human Research Ethics Committee (Medical):**

*If the following issues do not arise during the Theory of Change process, participants in the group ToC will be asked explicitly by the researchers:*

1. Is the depression screening questionnaire from the English PHQ-9 culturally accessible to psychiatric patients in South Africa?

1. Would using an electronic tablet constitute a cultural barrier for certain subgroupds of psychiatric patients?
2. How might cultural expressions and understandings of depression be made more accessible to South /African patients?
3. What linguistic barriers might be embedded within the current format of the patient PHQ-9 screening for depression to suit South African patients?

**References**

DE SILVA, M., LEE, L. & RYAN, G. May 2015. *Using Theory of Change in the development, implementation and evaluation of complex health interventions. A practical guide* [Online]. The Centre for Global Mental Health & Mental Health Innovation Network. Available:

[http://mhinnovation.net/sites/default/files/downloads/resource/MHIN%20ToC%20guidelines_May_2015.pdf [](http://mhinnovation.net/sites/default/files/downloads/resource/MHIN%20ToC%20guidelines_May_2015.pdf)Accessed 29/05/2016].

MENTAL HEALTH INNOVATION NETWORK 2014. Measuring the impact of funding portfolios: An introduction to Theory of Change approach. *In:* CENRE FOR GLOBAL MENTAL HEALTH (ed.). LSHTM.
